# A Bioeconomic Model for the Thoroughbred Racing Industry—Optimisation of the Production Cycle with a Horse Centric Welfare Perspective

**DOI:** 10.3390/ani13030479

**Published:** 2023-01-30

**Authors:** Kylie A. Legg, Erica K. Gee, Mary Breheny, Michaela J. Gibson, Chris W. Rogers

**Affiliations:** 1 School of Veterinary Sciences, Massey University, Palmerston North 4442, New Zealand; 2 School of Health, Victoria University of Wellington, Wellington 6140, New Zealand; 3 School of Agriculture and Environment, Massey University, Palmerston North 4442, New Zealand

**Keywords:** horse, racing, racehorse welfare, economics, Thoroughbred industry

## Abstract

**Simple Summary:**

The Thoroughbred racing industry is constrained by three major moderators: economics, horse biology and social licence to operate. The role these three moderators play in regulating the industry and the relative interaction of these components differs between racing jurisdictions. Most attention to date has been focused on addressing a single aspect of one moderator in isolation to improve industry performance. However, this review has identified the complexity of the interaction between these three moderators and the need to consider the overall effects of a change on each individual jurisdiction. Based on the data presented within this review, the authors propose that injury risk and wastage of racing horses need to be considered within the context of each jurisdiction operating as a unique bio-economic model.

**Abstract:**

The Thoroughbred racing industry faces new and competing pressures to operate within a modern, changing society. Three major moderators drive the focus and productivity of the industry worldwide: economic sustainability, horse biology and social licence to operate. This review proposes that despite the apparent homogeneity in the structure of racing across jurisdictions due to international regulation of the sport, there are significant differences within each jurisdiction in each of the three moderators. This creates challenges for the comparison of injury risk factors for racehorses within the industry across different jurisdictions. Comparison of the relative distribution of racing and gambling metrics internationally indicates that the Asian jurisdictions have a high focus on gambling efficiency and high economic return of the product, with a high number of starts per horse and the highest relative betting turnover. In contrast, the racing metrics from the USA have proportionally low racing stakes and fewer horses per race. These differences provide insight into the sociology of horse ownership, with a shift from the long-term return on investment held by most jurisdictions to a short-term transitional view and immediate return on investment in others. Wastage studies identify varying risks influenced by the predominant racing culture, training methods, production focus and environment within individual jurisdictions. Increasing societal pressure to maintain high racehorse welfare and reduce the negative impact of gambling poses fluctuating risks to each jurisdiction’s social licence to operate. Based on the data presented within this review, the authors propose that the use of a bioeconomic model would permit consideration of all three moderators on industry practice and optimisation of the jurisdiction-specific production cycle with a horse-centric welfare perspective.

## 1. Introduction

The horse occupies a unique place in modern society. The traditional or historical role of the horse was as a utility or production animal. During the latter half of the 20th century, the predominant use of the horse transitioned from a working animal to one solely for sport and recreation. With this change in role came a shift in the perception of acceptable welfare standards for horses, with most of society now identifying the horse as a companion animal or pet [1,2]. This contemporary framework places the racing industry in a unique position whereby they utilise the horse in a sport in which horses compete in races for the enjoyment and entertainment of a public audience and economic gain for industry participants.

Horse racing and breeding is an important industry in at least 71 countries, involving over half a million horses and offering over 3.3 billion EUR in prize money worldwide [3]. The major racing nations (ranked by number of racing horses) are presented in Table 1. Traditionally entrenched in society, horse racing has always been inextricably linked with gambling. Worldwide betting turnover exceeded 115 billion EUR in 2019, with over 105 million AUS in gambling revenue recorded from a single race (the Melbourne Cup, the premier race in Australia) [3,4]. Revenue streams to owners and trainers are heavily dependent on betting turnover, and thus the economic health of the industry is reliant on maintaining sufficiently high levels of gambling on each race [5,6].

Betting turnover, and thus the majority of industry revenue, is dependent on the number of races offered, the quality” of the race (the rating of the horses) and the number of participants within each race. These are constrained by the relative size of the racing population and the replacement rate of the racing population. In recent years, there has been increasing public scrutiny on the replacement rate and the issue of life after racing, i.e., what happens to horses once they leave the racing production system [7,8]. The replacement rate is influenced by the industry structure, which in turn is heavily constrained by the moderator “horse biology”. Like all athletes, horses require appropriate periods of training and recovery to optimise performance and minimise the risk of injury [9]. These periods of training (preparation) and recovery (spells) vary with the age of the horse and the racing focus [5]. The goal for racing administrators and participants is to balance the economic returns with the biological constraints, primarily through the frequency and intensity of racing. 

In recent times, the popularity of horse racing has diminished, with 70% of adult Australians professing little or no interest in the sport [10,11]. As society becomes increasingly urbanised, fewer people have any familiarity with horses or horse racing. Lack of familiarity with horses has been associated with an increasingly anthropomorphic view of the horse rather than one based on the horse’s ecological niche [2,12]. These changing social values, combined with new technologies that enable widespread dissemination of information, including the occurrence of injuries and catastrophic events on the racecourse, have contributed to increasing media attention and debate about the acceptable use of animals in sport [13]. The continued use of the Thoroughbred in racing requires critical social engagement to continue their social licence to operate [8]. These pressures for change from society, external to the racing industry, in turn, impact the structure, pattern and economics of racing. The magnitude and direction of change are dependent on the critical mass of each racing jurisdiction and its capability to respond. 

This review examines the three primary moderators within the horse racing industry (economics, horse biology and social licence). The need to consider all three moderators within a holistic framework when exploring changes in industry management or practice is explored. It is proposed that all three moderators interact differently within the separate racing jurisdictions, and thus, jurisdiction-specific socio/bioeconomic models are required to model the effect of industry change.

### A Bioeconomic Model of Racing and the Sustainability of Each Racing Jurisdiction

The racing industry cannot be defined as a simple biological process. There is a need to balance the economics of the industry with society’s expectations of the acceptable or ideal use of animals in both sport and production. These bioeconomic approaches are common within other animal industries, such as the dairy industry. Within the New Zealand dairy industry, the production system can be modelled to maximise output (milk yield) based on the relevant (and differing levels of) constraints peculiar to each region, using a framework of production economics. From these models, five farm system classifications have evolved, in simple terms, describing the relative contribution of grazed grass (low input) vs. imported feed types (high input) in relation to milk yield [14]. Though similar net returns are observed from each production system [15], the proportion of farms using each system depends on differing farmer goals, skills, knowledge, available resources and climate. In addition, the farm system used is heavily dependent on economic factors such as feed price and availability and milk pay-out as well as both financial and biological risk and animal welfare legislation [14,16,17]. These differing and competing pressures and constraints drive the choice of production system, to achieve the optimum level of least cost, optimum profit within the broader processes of socioeconomic change [17]. This modelling process can be translated to identify and quantify the economic, biological and social constraints and their interaction within the Thoroughbred racing industry. 

Population level research, both within and between racing jurisdictions, is vital to provide reliable independent data to inform discussion around the key community concerns that threaten the Thoroughbred industry’s social licence to operate. Improving welfare standards for horses as well as economic returns for industry participants is a key focus for the Thoroughbred industry, therefore, research to better understand both individual horse-level and industry-level determinants of a horse’s career duration and success is important. The key drivers of race programming, retirement, injury and economic returns for owners and trainers are important factors for investigation.

**Figure 1:**
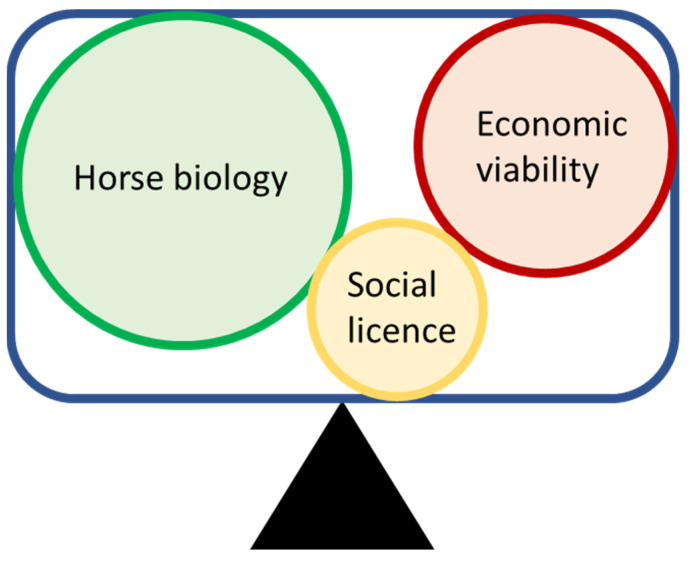
Conceptual schema of the bioeconomic constraints of a racing jurisdiction in equilibrium—alter one moderator and the whole system needs re-adjustment to establish equilibrium. Different jurisdictions would have different weightings for each of the three moderators.

A conceptual schema for the three major moderators within a racing jurisdiction is presented in Figure 1. In this conceptual model, much like the conceptual framework used to describe training volume in athletes (frequency, duration and intensity) [9], each jurisdiction has a unique bioeconomic constraint within which the three moderators interact agonistically against each other. Horse biology (and consideration of the racing environment) has the least opportunity to dramatically alter or influence the other two moderators. However, the differences in focus and social licence between racing jurisdictions create different pressures, weightings and interactions between each of the moderators, changing the optimum balance point for each jurisdiction to operate in equilibrium.

## 2. Economic Sustainability

The economic impact of the racing industry includes employment, export income and gambling, and in the US is estimated to be 26.1 billion USD [18]. Racing is one of the largest industries in Australia, contributing 0.5% to gross domestic product (GDP) [19] and in New Zealand, the racing sector is estimated to generate over 1.4 billion NZD (approximately 1%) in GDP [20]. Much of the revenue associated with horse racing is derived directly or indirectly via gambling. The strong co-existence of horse racing, gambling and economic viability has resulted in strong external (national or federal government) control and robust internal industry control of racing and processes. 

Externally, the international racing industry appears relatively homogeneous with the structure and regulation of racing for the major racing jurisdictions coordinated via the International Federation of Horse Racing Authorities (IFHRA) [3]. However, summary data on the number of races, prize money offered and the level of wagering on races across the three major racing regions of Asia, Europe and the Americas reflect significant differences in the level of racing and the economic pressures within even these broad regions (Table 1). As an example, the Asian region holds 40% of all flat races, accounting for 56% of the total stakes money on offer, and is responsible for over 60% of all money gambled on horse racing worldwide. In contrast, the Americas hold almost the same number of races (39% of all flat races) but offer only 29% of total stakes money and are responsible for only 10% of the total international wagering [3]. The relatively lower stake money percentage on offer in the USA reflects the large number of races that are claiming races (reported in some states to represent up to 54% of races) [21]. A claiming race is a type of horse race in which the horses are all for sale at a specified “claiming” price until shortly before the race and generally includes the lowest quality of horse, with the lowest stakes money of all race types [21]. This type of race alters the dynamics of horse ownership and reduces the time frame of interest for the horse owner from a long-term commitment with a focus on individual owner-trainer relationships, to a short-term, transitional perspective of horse ownership. Claiming races are additionally associated with a higher risk profile for catastrophic musculoskeletal injury [22], indicating that economics may be given a larger weighting than “horse biology” within the Americas.

There are large differences between both broad racing regions and individual jurisdictions in racing population size and the number of horses competing in a race. As field size increases, there are more betting opportunities and funds gambled on the race, with ≥12 horses per race needed to optimise the betting pool (total money gambled) in any given race. Optimisation of the betting pool is important, as a percentage of all money gambled is returned to the industry in the form of race stakes, providing the revenue stream for owners, trainers and jockeys [6,23]. 

The Australasian region has a similar sized racing population to the Americas (39% of the worlds racing population of horses), but the average number of starters per race is higher in Australasia, with 10.2 horses per race, compared to a mean of 7.4 horses per race in the Americas. Asia and Europe have a lower total number of horses (18% and 14%, respectively) but maximise betting opportunities with a mean of 11.1 and 10.7 horses per race, respectively (Table 1). These metrics demonstrate the dependence of most jurisdictions on optimising gambling opportunities, whereas the Americas are unique with their focus on immediate returns from horse ownership.

The categories of betting turnover per race start and per horse in the industry provide some comparative metrics on the relative ability of the different industries to provide economic return on their respective horse and racing populations. The betting turnover relative to the number of starters, and perhaps more so with the betting turnover relative to the number of horses in the industry, clearly demonstrates that some jurisdictions such as Hong Kong, Great Britain and Korea are very effective at optimising gambling opportunities (Table 1). 

Hong Kong and Singapore are unique populations with a high economic return of product, averaging 12 horses per race, with an average of 7.7 starts for a horse each year. Europe has the lowest frequency of horses racing, with 4.9 starts per horse per year. However, the European flat racing season is mainly truncated to the summer months (approximately 8 months, April to November), whereas in Hong Kong, racing is conducted for 11 months of the year, with a compulsory 1 month break in racing during August. Therefore, the shortened season in Europe provides fewer opportunities for horses to race. In the USA and Australasia, racing is conducted year-round, with 5–6 starts per horse per year. This difference in race starts potentially reflects industry constraints from the environment (e.g., duration of the racing season) as well as cultural traditions or training practices. However, the “off-season” may have other industry benefits, such as providing a yearly “revitalisation” of the industry as fans, horses, and industry participants start each season after the rest period fresh and eager to succeed. These breaks may additionally help to increase and revitalise interest in gambling, and thus, maximise betting turnover, as evidenced by the high betting turnover relative to number of horses racing in both Asia and Great Britain.

### Breeding Sector

Most racing jurisdictions have some form of vertical integration where the domestic breeding sector generates enough horses to meet the replacement rate within the racing industry. Between jurisdictions, there is considerable variation in the number of foals produced per year relative to the domestic racing population, which reflects the differences in production focus. Some countries breed only for domestic supply, and others have a large export focus for the racing product (Table 2). The jurisdictions that have a domestic breeding and racing focus have a foal production rate of approximately 30–40% of their current racing population per year. This value aligns with the annual turnover of horses within a racing population of between 20–40% [5,24].

There are also some unique racing jurisdictions, such as Hong Kong and Singapore, with no domestic breeding programme and are dependent on importing 100% of their Thoroughbreds from overseas nations (mainly Australasia). This contrasts with self-supporting nations such as the Americas and Japan that largely maintain their racing population through foals born domestically. Ireland is a highly export-focused nation, breeding more than twice the required replacement rate to sustain domestic racing [25]. New Zealand is essentially a hybrid system, with both a self-sustaining breeding sector and a large export-focused breeding sector [26]. These differences in production focus between jurisdictions may alter both the economic focus and the weighting of social concerns regarding wastage and overproduction of raw material (youngstock/foals).

**Table 2:** Import and export data estimated for major racing nations for the 2019 season.

Country	Imports per Year	Imports per Race Population	Exports per Year	Exports per Race Population	Number of Foals Produced	Foals per Race Population
USA	808	2%	2326	5%	19,925	44%
Australia	2515	7%	1681	5%	12,944	37%
Japan	453	2%	43	0%	7368	30%
Great Britain	1965	17%	1553	13%	4748	41%
New Zealand	410	9%	1771	37%	3489	73%
Ireland	310	7%	4049	95%	9295	219%
Hong Kong	420	100%	-	-	0	0%
Singapore	-	100%	-	-	0	0%

Sources: [3,27,28,29,30,31].

## 3. Horse Biology

The genotype and phenotype of the Thoroughbred racehorse is relatively homogeneous due to over 300 years of selection for racing ability from a relatively narrow genetic base [32]. There are some subtle differences between jurisdictions in the early foal-rearing environment. Intervention studies have identified that some of these differences in foal rearing may positively contribute to the growth and development of the musculoskeletal system and thus reduce the subsequent injury risk profile [33]. However, the difficulty in obtaining precise measures of early life exercise at a population level has precluded refinement of estimation of the effect size contributed by the early production environment. 

In 1982, Leo Jeffcott and colleagues published the first epidemiological study to examine wastage within the Thoroughbred racing industry [34]. Since this publication, there has been approximately 40 years of attention focused on quantifying different aspects of wastage; from examination of the whole supply chain [35] through to very specific race-level risk factors for injuries such as dorsal metacarpal disease in 2-year-old racehorses [36]. A number of authors have attempted to examine more holistic measures of race-day injury, including veterinary events, stipendiary stewards reports and failure to finish outcomes [37,38,39]. These metrics are useful for the quantification of negative events during or associated with racing within a jurisdiction. They provide, to a certain extent, an indication of the robustness of specific industry regulation and identification of incidents, but to date, these have rarely been used to provide comparative metrics across jurisdictions [22]. 

Welfare concerns for the Thoroughbred racing industry focus not only on the catastrophic injuries that may be reported within the media but also on the loss of horses from the industry [8,40]. When the industry is examined as a supply chain, horses that do not enter racing are often included in the group “wastage”. However, this can be a gross oversimplification of the opportunities for progression for a foal born and entry into racing. One proposal from groups antagonistic to racing is that the industry should breed only the number of foals equivalent to that required to replace the racing population. This proposal ignores that the horse is a biological organism, and thus subject to natural variation within the population. There is also the confounding factor that racing, as a sport, aims to identify the elite from within each cohort, so they may, in turn, contribute to the improvement of the next generation. 

When examined as a supply chain, there are similar rates of attrition in racing and other equestrian sports such as show jumping, dressage and eventing. Within any given year cohort, about one-third to one-half of horses born will not enter sport or racing [34,35]. Of the remaining two-thirds of the foal crop, one-third will retire or withdraw from training due to voluntary reasons (often lack of ability), one-third will withdraw due to involuntary reasons (predominantly musculoskeletal injury) and one-third will remain within the production system [34,35,41,42]. This uniformity of attrition between equestrian disciplines emphasises the consistent expectation of an inherent redundant component of the biological population (horse) to have the opportunity, the talent and the orthopaedic health to have a sporting career. Similar key (biological) limitations in the progression of athletes have been identified in human sport [43], reflecting the need to describe and document the underlying biological variation to be able to optimise flow of product (athlete or horse) through the system and target the individuals with the greatest opportunity for success.

### 3.1. Voluntary Losses

Those horses which do not enter racing or are voluntarily retired are collectively labelled “voluntary losses”. Some of the loss of horses before entering training, and voluntary loss within training, can relate to industry structure and economics. The majority of flat racing is focused on horses aged 2 to 5-years old [5,7,44]. However, in Australia, 5% of the annual racing population of horses have only one race start [40]. This figure is higher in New Zealand, with a consistent 14% of horses annually having only one race start [5]. Due to the export focus of the New Zealand industry, many of these horses with one race start are believed to be exported at the beginning of their careers. 

These metrics imply that there is early identification within the industry of horses who lack ability or are unsuited to the sport. Voluntary retirement allows for the option of early repurposing in other disciplines [45], with voluntary rather than involuntary retirees having increased odds of repurposing as performance horses [46]. This may represent a self-supporting industry mechanism to improve horse welfare and economic benefit by not racing individuals that obviously, at initial screening, do not have the physical or mental characteristics to permit them to succeed within the racing or sport industry.

### 3.2. Involuntary (MSI) Losses

For those horses that enter training, musculoskeletal injury (MSI—fracture and soft tissue injury) is the most common reason for involuntary loss from the horse racing industry, accounting for 80% of involuntary interruptions to training and 25% of horses exiting from the industry [47,48]. For many jurisdictions, there has been quantification of the prevalence and, in some cases, quantification of risk factors for a range of case definitions from condylar fracture of the metacarpal and metatarsal bones [49] to all MSI [50,51]. To date, over 300 different factors have been examined as risk factors for catastrophic musculoskeletal injury [22]. These incidents represent perhaps the most emotive and tragic incidents that occur in racing and could be considered the most industry-threatening incidents due to compromised social license [8]. 

The risk factors for MSI, and those for other injuries, and even the holistic measures of racing industry integrity (such as failure to finish a race) can be broadly grouped into horse-, race-, management- and environment-level factors. Despite the homogeneity of racehorse genotype and phenotype, there are subtle differences between jurisdictions in horse-level risk factors (such as age and sex). The interaction of the racing jurisdiction (in this case, country) on risk factors for catastrophic musculoskeletal injury was elegantly demonstrated within the meta-analysis conducted by Hitchens, Morrice-West, Stevenson and Whitton [22]. This study highlighted the confounding effect that the production system (jurisdiction) has on the identification and estimation of the level of risk of specific risk factors. These production interactions relate directly to how the horses are trained and raced, as well as environmental conditions such as racing track surface type and permitted medications.

In most jurisdictions, Thoroughbred racing is regarded as “drug-free”, with extensive restrictions on permitted medications and administration of medications relative to race day [3]. However, in the USA, there are differences in medication control between states, with some states permitting the use of furosemide (a diuretic used to reduce the risk of exercise-induced pulmonary haemorrhage), phenylbutazone (a non-steroidal anti-inflammatory drug) or intraarticular injection of corticosteroids (anti-inflammatory medicine), all of which are prohibited at the time of racing in most jurisdictions. These practices have been the source of recent tension between the USA racing regulators and the IFHRA. Use of certain medications in racing are believed to be a risk factor for MSI [22], with horses who raced with a declared prerace administration of phenylbutazone 50% more likely to sustain a fatal or nonfatal MSI than those racing without a prerace administration of the non-steroidal anti-inflammatory [52]. The effect of drug use is, however, confounded by the reason the drug was required in the first place. For example, a horse requiring pain medication may have an undiagnosed hairline fracture that predisposes them to a catastrophic fracture in-race.

Horse racing is conducted mainly on turf or synthetic (all-weather) surfaces, apart from in the USA and Canada, where 75% of tracks are dirt or sand based [28]. The majority of racing (73–100%) in Great Britain, South Africa, NZ, Ireland and Hong Kong is on turf, with synthetic tracks more prevalent in Singapore [53,54]. In most racing jurisdictions, racetracks are flat, oval-shaped and the horses race in one direction throughout the race. However, in Europe, racetracks are of varying shapes and sizes and may include undulations and a combination of left- and right-hand turns within one track. Track surface, consistency and changes in the horses leading leg alter the level of biomechanical load on the horse with each stride. This variation in loading pattern is elegantly demonstrated in the differing incidence rates and types of MSI observed on different track surfaces and track shapes [55,56]. 

A number of studies have shown a positive association of cumulative amounts of high-speed exercise with fracture risk and MSI [48,57,58,59,60,61,62]. However, more than half of Australian Thoroughbred training programmes exceed previously reported risk levels for MSI with high volumes of gallop work, but their fracture risk (in races) is lower than reported in other jurisdictions [22,63,64]. The complex relationship between training intensity, speed and rest periods [9,65] suggests conflicting mechanisms of injury related to the accumulation of bone damage [22]. These mechanisms may relate directly to training and management practice differences between jurisdictions, namely: well-adapted bone after an intense period of training, or poorly adapted bone at relatively low levels of training intensity [22]. There are large differences in reported training loads between jurisdictions (Table 3). Based on the published data, there appear to be distinct differences in the training philosophy between Europe and Australasia. In Europe, the training focus for horses is to minimise injury through large quantities of long slow distance training to obtain maximal fitness [64]. In contrast, training in Australasia appears to have greater specificity, with higher gallop volumes and less slow work (minimal training load) than observed with European horses [9,63,66].

Differences in the average career length of Thoroughbred racehorses may change the risk profile of horses within a jurisdiction. However, there are few comparative metrics on the average career length of Thoroughbred racehorses. Average career lengths for horses in New Zealand have been reported as 15 months, 18 months in Turkey and 21 months in Australia [5,72,73]. A recent trend reported in New Zealand has been a right shift in the age profile of the racing population due to the delay in retiring older horses, believed to be an industry-level compensation for a reduced supply of new (younger) horses into the racing industry [5,26,38]. This provides an interesting interaction of older horses, greater frequency of racing and greater accumulated load cycles (racing and training) which would be expected to alter the population risk profile for MSI.

## 4. Social Licence

Social licence considers the role of wider society in sanctioning or censuring an activity. It can differ according to the reference frame in which the sport is presented (internal industry or external acceptance) [2] and is also moderated by the economics of the society providing the reference frame [74]. Horse racing has been increasingly controversial over the last decades, mostly in relation to whip use as well as injuries and fatalities, particularly from jumps racing [2,13,75,76,77]. More recently, industry and public attention has shifted focus towards the end of racing career life for horses and associated “wastage”, particularly in response to media commentaries focusing on the Australian Thoroughbred racing industry and similar exposés in Europe [10,78].

Levels of public interest in racing and the public’s ease of access to and interaction with racing as a sport are important factors in maintaining or undermining social license. Changes in the technological environment (e.g., online gambling vs. live attendance) may intersect with the social licence aspects in terms of accessibility to the industry and a sense of commitment to its continuation. In horse racing, the social norms that people place a bet on feature races such as the Melbourne Cup in Australia or the Grand National in the UK may build a connection with the sport and, consequently, interest in its continuation [11,79]. However, this visibility is fraught as it also means any catastrophic injury is broadcast to a large international audience in real-time, sparking public concern. Industry secrecy is common when contentious animal use practices are employed, however, transparency of practices is increasingly expected from public-serving industries to maintain their social license to operate [80]. 

**Figure 2:**
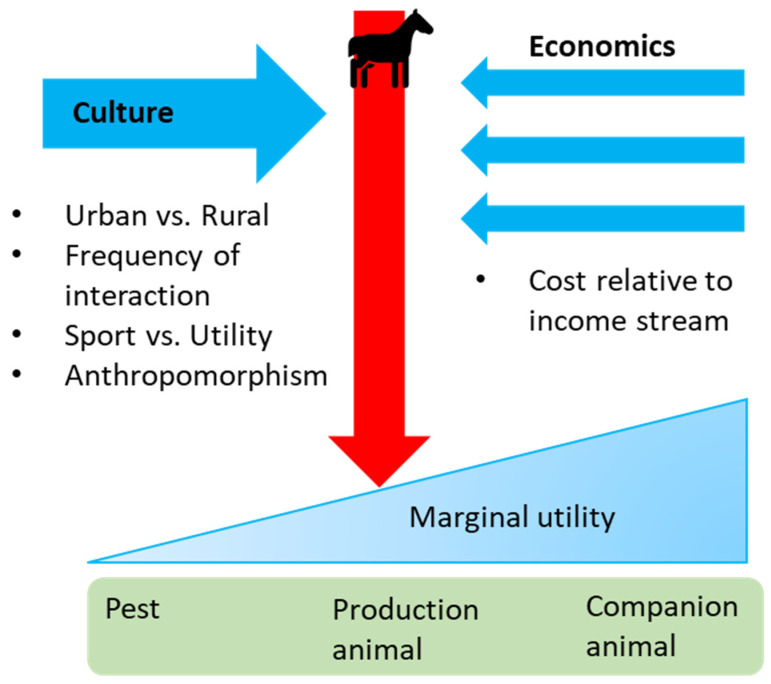
Conceptual schema of a sliding scale of marginal utility (worth) of the horse. Where the horse sits on the scale may be influenced by various perspectives from different aspects of society, which in turn alters the social licence of the use of the horse in the racing industry.

An increase in public awareness of animal welfare issues changes the perception of what may constitute acceptable loss in Thoroughbred racing. A catastrophic musculoskeletal (MSI) rate of 1.2 per 1000 race starts [22] may be viewed by the industry as an acceptable rate of loss in this highly demanding athletic sport, whereas any death may be viewed as unacceptable by animal welfare advocates [2]. These deaths can be widely disseminated by animal welfare outlets to the public to highlight the harm caused by the Thoroughbred racing industry. The Thoroughbred racing industry has recognised a need to reduce horse injury and loss to meet the social licence to operate effectively within a changing society. In modern, increasingly urban culture, a horse may be viewed variously as a production animal (product of the racing industry), a highly conditioned athlete, or even as a pet or companion animal by different sectors of society [2,81] (as shown in Figure 2). The specific context of each jurisdiction influences the social perception of the horse; for example, the use of claiming races in the USA, may alter the perception of the racehorse from a prized and pampered athlete to a return on investment, with a corresponding lower marginal utility [2].

Addressing the animal welfare concerns of Thoroughbred horse racing may be viewed as a niche concern. For most people, the heavy regulation or abolition of horse racing is unlikely to require any change in habits in the way that changes in farming or companion animals might alter patterns of general consumption or personal behaviour. However, changes for the participants within the industry could come with large financial and emotional costs.

Complicating the social license of Thoroughbred horse racing is the industry’s integration with and reliance on gambling. There is widespread recognition that gambling causes social harm, and only the offsetting of social harm with social good can justify its existence. Gambling concerns provide a different focus for undermining Thoroughbred horse racing. These are contradicted by libertarian principles of individual choice, however, there are arguments for gambling harm initiatives to include socio-cultural approaches which may impact on the broad culture of gambling within the racing industry [79]. This would have major consequences for the industry, with their heavy reliance on gambling income to sustain the economics of the industry. Some jurisdictions, compensate for any negative impacts of gambling in society through charitable donations to the community on projects such as youth development, emergency and poverty relief and care of the elderly, becoming a leader in charity donation in Hong Kong [82].

## 5. Balancing Model Factors

**Figure 3:**
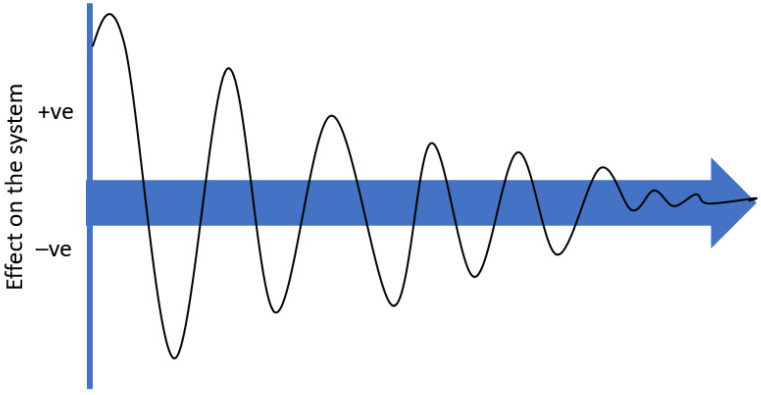
Theoretical depiction of the effect from a change to a single industry moderator, causing the overall effect on the entire system to oscillate until equilibrium is re-established between all three industry moderators (economics, horse biology and social licence).

The racing industry is not a simple linear process, as can be seen by the biology of fracture risk and quadratic relationship with high-speed exercise [64]. Single changes to improve the effect on one industry moderator in response to risk factors considered in isolation may negatively (or positively) affect different components of each of the other two industry moderators. This may create an oscillation in the entire system, as the three moderators fluctuate with competing pressures until the industry re-establishes an equilibrium. The overall effect of industry response to an isolated change is theoretically modelled in Figure 3. Therefore, simply acting on risk factors (or reducing stress points) identified in isolation, some of which may not be practically modified, may not result in an overall benefit to the horses within the system. 

An example of the difficulties faced in racing industry optimisation has been observed in Keeneland, USA. In 2014 the synthetic racetrack was replaced with dirt, seemingly due to industry pressures from racehorse trainers and the wish to reduce the costs of track maintenance. This was despite a fatality rate on the previous synthetic track half that was reported on dirt tracks. By improving the economic factor, there was an adverse effect on horse biology with increased odds of catastrophic MSI. This impacted the industry’s social license to operate due to public outrage for the apparent lack of welfare concern by industry participants. This highlights the different weights given to each of the three different moderators in different jurisdictions.

Determination of the acceptable level of risk, injury and wastage within the system may become the dominant driver of the processes within the industry. Proponents of horse welfare may argue that no risk is the only acceptable level, however, this would be an impossibility within any biological system without the complete cessation of horse racing. Within every biological system, there must be loss and replacement. However, the focus of sustainability of the Thoroughbred industry may need to shift from viewing the horse as part of a production system, to optimisation of the system with a horse-centric welfare perspective [83].

A bioeconomic model could provide an indication of what could be done to balance system efficiency with horse welfare to meet the socioeconomic factors peculiar to each jurisdiction. In agricultural systems, these bioeconomic models are extensively used to predict changes in farm-level systems to the biology of the system and the flow-on effects. It would combine the physiological limitations of the horse (e.g., the frequency and number of starts horses can safely complete) with the optimal economic returns to the industry (e.g., the number of horses required to race), and the welfare concerns (e.g., what poses least risk to the horse), to give an indication of the optimum biologically feasible number of horses required to race within the system. Necessary biological constraints would include both training and racing metrics such as spell length, preparation length, number of starts and days between races to determine the optimal physiological preparatory load. However, each factor does not operate independently of the others but rather interacts, resulting in fluctuating pressures on the industry. Therefore, it is suggested that the industry is modelled as an open ecological system, following the approach used for complex questions such as climate change and dairy farm systems [14,17,84]. To proactively instigate measures that improve the welfare of the horse, the focus should be on optimising the system as a whole, rather than focusing on reducing a single aspect of wastage in isolation.

## 6. Conclusions

Thoroughbred racing as a sport and industry is constrained by the requirement for economic sustainability, horse biology and obligations to meet society’s expectations in relation to social licence to operate. To date, much attention has focused on addressing these three moderators, and components of these moderators in isolation. This review has identified the complexity of the international racing industry, the interaction of economics, horse biology and social licence to operate, and the need to consider the separate racing jurisdictions as open ecological systems. The use of a bioeconomic model would permit consideration of the moderators on industry practice and optimisation of the production cycle with a horse-centric welfare perspective. 

## Figures and Tables

**Table 1:** Summary data of racing and economic return levels for the major racing nations in 2019. Source: International Federation of Horse Racing Authorities [3].

Country	No. of Races	No. of Horses Racing	Starts	Av. Starts per Horse	Av. Number of Horses per Race	Stakes (Euros)	Average Prize Money per Race	Betting Turnover (Euros)	Betting Turnover per Starters	Betting Turnover per Horse
USA	36,066	44,887	271,415	6.1	7.5	€842,570,286	€23,362	€9,825,137,400	€36,200	€218,886
Australia	19,276	34,939	181,264	5.2	9.4	€456,662,546	€23,691	€18,254,320,764	€100,706	€522,463
Japan	16,444	24,595	178,835	7.3	10.9	€964,829,380	€58,674	€29,405,021,378	€164,425	€1,195,569
Great Britain	6366	11,527	59,974	5.2	9.4	€130,737,028	€20,537	€17,996,089,757	€300,065	€1,561,212
Argentina	5613	11,122	56,015	5.0	10.0	€23,558,402	€4197	€80,106,969	€1430	€7203
France	4918	9926	51,167	5.2	10.4	€121,911,271	€24,789	€8,825,473,709	€172,484	€889,127
Turkey	5579	6234	55,137	8.8	9.9	€67,987,230	€12,186	€1,042,096,197	€18,900	€167,163
South Africa	2955	5760	31,159	5.4	10.5	€17,367,097	€5877	€146,894,903	€4714	€25,503
New Zealand	2482	4759	26,225	5.5	10.6	€34,539,955	€13,916	€361,897,701	€13,800	€76,045
Canada	3135	4726	22,576	4.8	7.2	€67,825,831	€21,635	€945,598,832	€41,885	€200,084
Brazil	3039	4648	-		-	€13,024,200	€4286	€58,755,090	-	€12,641
Chile	4978	4338	58,320	13.4	11.7	€24,295,804	€4881	€234,923,199	€4028	€54,155
Ireland	1239	4244	15,038	3.5	12.1	€34,291,000	€27,676	€4,750,880,837	€315,925	€1,119,435
Morocco	2463	3874	23,920	6.2	9.7	€11,970,478	€4860	€669,879,829	€28,005	€172,917
Uruguay	1640	3842	16,832	4.4	10.3	€10,039,718	€6122	€19,215,838	€1142	€5002
India	2514	3760	24,258	6.5	9.7	€14,808,562	€5890	€250,258,051	€10,317	€66,558
Korea	1893	3716	20,701	5.6	10.9	€163,995,920	€86,633	€4,953,444,364	€239,285	€1,333,004
Hong Kong	828	1398	10,227	7.3	12.4	€152,781,158	€184,518	€13,899,836,251	€1,359,131	€9,942,658
Singapore	772	1109	8941	8.1	11.6	€31,622,789	€40,962	€681,298,639	€76,199	€614,336

**Table 3:** Reported training and racing volumes per month for horses from major racing populations.

Country	Reference	Horse Age	Sample Size (Horses)	Canter (m/Month)	Gallop (m/Month)	Length of Spell (Weeks)	Starts per yr
USA	[67]	2 yr-old	226	28,400	1000	-	
	[68,69]	>2 yrs	6–618	23,004	1800–2640	-	6.1
Australia	[63]	2 yr-old	~287	60,000	6400	-	
		>2 yrs	~1433	73,200	9600	6.3	5.2
Great Britain	[36]	2 yr-old	335–647	16,800–18,940	960–1520	-	
	[64]	>2 yrs	860–1176	26,800–37,240	2800–4380	-	5.2
New Zealand	[5,70]	2 yr-old	7	63,200	4800	6.5	2
	[5,71]	>2 yrs	53–30,254	68,796–76,200	8408	13	5.5

## Data Availability

Data used is available from the International Federation of Horse Racing Authorities (IFHRA).
